# Role of Nutraceuticals in Counteracting Inflammation in In Vitro Macrophages Obtained from Childhood Cancer Survivors

**DOI:** 10.3390/cancers16040714

**Published:** 2024-02-08

**Authors:** Alessandra Di Paola, Maria Maddalena Marrapodi, Elvira Pota, Rosa Colucci Cante, Deeksha Rana, Giulia Giliberti, Giuseppe Di Feo, Shakeel Ahmed, Domenico Roberti, Roberto Nigro, Francesca Rossi, Maura Argenziano

**Affiliations:** 1 Department of Woman, Child and General and Specialist Surgery, University of Campania “Luigi Vanvitelli”, 80138 Naples, Italy; alessandra.dipaola@unicampania.it (A.D.P.); mariamaddalena.marrapodi@unicampania.it (M.M.M.); elvira.pota@gmail.com (E.P.); giuseppe.difeo.bio@gmail.com (G.D.F.); domenico.roberti@unicampania.it (D.R.); maura.argenziano@unicampania.it (M.A.); 2 Department of Industrial Engineering, University of Niccolò Cusano, 00166 Rome, Italy; rosa.coluccicante@unina.it; 3 Department of Experimental Medicine, University of Campania “Luigi Vanvitelli”, 80138 Naples, Italy; deeksha.rana@unicampania.it (D.R.); giulia.giliberti@unicampania.it (G.G.); shakeel.ahmed@unicampania.it (S.A.); 4 Department of Chemical, Materials and Industrial Production Engineering, University of Naples Federico II, 80125 Naples, Italy; roberto.nigro@unina.it

**Keywords:** childhood cancer survivors, inflamm-aging, macrophages, iron metabolism, nutraceuticals

## Abstract

**Simple Summary:**

Chemotherapy and radiotherapy exposure cause a long-lasting low-grade inflammatory state that is responsible for inflamm-aging, with the early onset of different chronic diseases that usually appear later in life, i.e., cardiac damage, bone mass loss, and cellular senescence. To prevent inflamm-aging, there is a need to find new possible therapeutic strategies, targeting cells involved in the immune processes. Macrophages are mononuclear cells involved in immune and inflammatory responses that play a crucial role in iron metabolism. In addition, inflammation causes an increase in intracellular iron concentration, responsible for inducing inflammation as well. Our results show that CCS patients’ macrophages are characterized by an increase in activated phenotype markers (M1) (inflammatory ones). We propose macrophages and iron metabolism as novel therapeutic targets to counteract inflamm-aging, and suggest the employment of different nutraceuticals (resveratrol, curcumin, and oil-enriched lycopene) to avoid other side effects of toxic regimens and to contain inflammation.

**Abstract:**

The advancement of anti-cancer therapies has markedly improved the survival rate of children with cancer, making them long-term childhood cancer survivors (CCS). Nevertheless, these treatments cause a low-grade inflammatory state, determining inflamm-aging and, thus, favoring the early onset of chronic diseases normally associated with old age. Identification of novel and safer therapeutic strategies is needed to counteract and prevent inflamm-aging. Macrophages are cells involved in immune and inflammatory responses, with a pivotal role in iron metabolism, which is related to inflammation. We obtained macrophages from CCS patients and evaluated their phenotype markers, inflammatory states, and iron metabolism by Western blotting, ELISA, and iron assays. We observed a strong increase in classically activated phenotype markers (M1) and iron metabolism alteration in CCS, with an increase in intracellular iron concentration and inflammatory markers. These results suggest that the prevalence of M1 macrophages and alteration of iron metabolism could be involved in the worsening of inflammation in CCS. Therefore, we propose macrophages and iron metabolism as novel therapeutic targets to counteract inflamm-aging. To avoid toxic regimens, we tested some nutraceuticals (resveratrol, curcumin, and oil-enriched lycopene), which are already known to exert anti-inflammatory properties. After their administration, we observed a macrophage switch towards the anti-inflammatory phenotype M2, as well as reductions in pro-inflammatory cytokines and the intracellular iron concentration. Therefore, we suggest—for the first time—that nutraceuticals reduce inflammation in CCS macrophages through a novel anti-inflammatory mechanism of action, modulating iron metabolism.

## 1. Introduction

In recent years, the advancement of anti-cancer treatments has improved the survival rate of children with cancer, making them long-term childhood cancer survivors (CCS) [1,2]. Despite encouraging increased survival in pediatric cancer, CCS often experience compromised health status as a late effect of anti-cancer treatments [1,2,3,4,5]. Chemotherapy and radiotherapy are accountable for the onset of late complications which hinder normal aging processes, affecting multiple organ systems and leading to a premature risk of age-related chronic diseases, frequent hospitalizations, and early mortality during adulthood [2,6,7,8]. This condition is also known as frailty [2,9,10].

These treatments lead to a low-grade chronic inflammatory state followed by an accelerated “aging” process—called “inflamm-aging” [2]. Treatments like chemotherapy and radiotherapy lead to the production and activation of reactive oxygen species (ROS) and reactive nitrogen species (RNS), which in turn are responsible for an increased inflammatory state. ROS and RNS induce DNA damage; pro-inflammatory cytokine production; and activation and proliferation of lymphocytes and macrophages, the main cells involved in immune and inflammatory responses [2].

The activation of lymphocytes and classically activated macrophages (M1) leads to a further increase in cytokine release, which participates in the impairment of inflammation and immunosuppressive functions of these cells [2,11,12].

Macrophages are mononuclear immune cells mainly involved in inflammatory responses [2,11]. They are present in two different activation phenotypes: M1 macrophages and M2 alternatively activated macrophages [13,14,15]. The M1 phenotype shows pro-inflammatory, anti-cancer, and antimicrobial functions. It is activated by tumor necrosis factor-α (TNF-α), interferon-γ (IFN-γ), and bacterial lipopolysaccharide (LPS), and determines the release of elevated levels of pro-inflammatory cytokines, such as TNF-α, interleukin (IL)-6, IL-1β, and nitric oxide synthase (iNOS). Instead, M2 macrophages have anti-inflammatory and immunosuppressive activities; they are involved in the release of anti-inflammatory cytokines, such as IL-10 and transforming growth factor-β (TGF-β), and are activated by IL-4, IL-10, IL-13, and the PI3K-Akt-signaling pathway mTOR [13,14,15].

It is well known that macrophages play a pivotal role in iron homeostasis [16,17]. In particular, M1 macrophages show increased iron internalization, expressing prominent levels of divalent metal transporter-1 (DMT1); M2 macrophages are involved in iron release [13,18,19]. Iron is a principal element in several metabolic processes, and the impairment of its homeostasis induces cellular damage [18,19]. Particularly, intracellular iron accumulation increases ROS production and the release of pro-inflammatory cytokines. There is a close correlation between inflammation and iron. Pro-inflammatory cytokines, in particular IL-6, increase hepcidin production and activation [20,21]. Hepcidin is a key hormone involved in iron metabolism; it binds to the only known transporter responsible for iron release, ferroportin (FPN-1), and leads to its degradation, also resulting in an increase in intracellular iron concentration [16]. Iron accumulation in macrophages alters their activity, contributing to the alterations to the inflammatory state [13,18,22]. Therefore, it could be interesting to use iron and its metabolism as therapeutic targets to counteract inflammation, which is a common condition in CCS.

In recent years, much interest has been directed to the use of nutraceuticals in different pathological conditions [23,24]. Nutraceuticals are compounds obtained from nutrients, herbs, and foods which can be used not only for nutritional support, but also for their new and emerging therapeutic properties [25,26]. Indeed, it is reported that these molecules can show different therapeutical functions, exerting anti-inflammatory, antioxidant, and anti-cancer effects. They stimulate the immune system against several pathological conditions, like inflammatory diseases, infections, cancer, and gastroenterological and neurological diseases [23,25,27,28,29]. In our study, we focused on the use of nutraceuticals, in particular resveratrol (Res), curcumin (Cur), and oil-enriched lycopene (OE-Lyc), which are well known for their anti-inflammatory responses.

Res, a compound synthetized by plants after stress stimuli, is a nutraceutical known for its several protective properties [29,30,31,32,33]. It shows anti-inflammatory, antioxidant, and anti-aging effects both in vitro and in vivo [34,35,36,37,38]. Cur is another natural compound which has been demonstrated to be a nutraceutical with anti-inflammatory properties [39,40]. It induces anti-inflammatory effects by modulating several pathways involved in inflammation [39,41,42,43,44,45,46]. Lyc is a carotenoid derived from tomatoes which exerts antioxidant and anti-inflammatory properties. It has been proven that its administration is useful for the management of different inflammation-associated diseases, such as cardiovascular diseases, obesity, and cancer [47,48,49,50,51].

Considering the interesting anti-inflammatory properties of Res, Cur, and Lyc and their known capabilities to regulate macrophage activity, the purpose of our research study is to evaluate the effects of these compounds on macrophages obtained from CCS patients. Thus, we propose a novel mechanism of action of these nutraceuticals, suggesting iron as a possible therapeutic target.

## 2. Materials and Methods

### 2.1. Source of Macrophages

Macrophages were obtained from the peripheral blood of 20 CCS patients (median age: 14 ± 4 years old) and 10 healthy donors (median age: 12 ± 4 years old), namely, subjects without any inflammatory, infectious, or oncological diagnoses. Blood from the CCS patients was drawn between 5 and 13 years after stopping therapy. Both the CCS and healthy donors were enrolled at the Department of Women, Child, and General and Specialized Surgery of the University of Campania “Luigi Vanvitelli”. All procedures in this study were executed in accordance with the Helsinki Declaration of Principles; the Italian National Legislation; and the Ethics Committee of the University of Campania Luigi Vanvitelli, which formally approved the study (Ethical Committee id code: 266, approved on 18 September 2020). Written informed consent was obtained from the participants’ parents.

### 2.2. Macrophages Primary Cultures

Macrophages were obtained from peripheral blood mononuclear cells (PBMCs). PBMCs were isolated by means of density gradient centrifugation (Ficoll 1.077 g/mL; Lympholyte, Cedarlane Laboratories Ltd., Uden, The Netherlands); diluted at 1 × 10^6^ cells/mL in α-Minimal Essential Medium (α-MEM) (Lonza, Verviers, Belgium) supplemented with 10% fetal bovine serum (FBS) (Euroclone, Siziano, Italy), 100 IU/mL penicillin, and 100 g/mL streptomycin and L-glutamine (Gibco Limited, Uxbridge, UK); and plated in 24-well Cell Culture Multiwell. To obtain fully differentiated human macrophages, the PBMCs were cultured for 15 days (about 2 weeks) in the presence of 25 ng/mL recombinant human macrophage colony-stimulating factor (rh-MCSF) (Peprotech, London, UK). The culture medium was replaced twice a week. Cells were cultured at 37 °C in a humidified atmosphere with 5% CO_2_. Macrophages were treated with Res, Cur, and OE-Lyc for 24 h after complete differentiation. Cells were then harvested for protein extraction, and cell culture supernatants were collected to perform iron assays and ELISA.

### 2.3. Drugs and Treatments

Res and Cur were both purchased from Sigma Aldrich (St. Louis, MO, USA) as powders. Res was diluted in sterile water to obtain the two final dilutions used on the CCS macrophage cultures: 10 µM and 20 µM [52]. Cur was instead dissolved in pure ethanol and used in vitro at the following concentrations: 5 µM, 10 µM, and 20 µM [53]. Oil-enriched lycopene (OE-Lyc) was obtained using an extraction process in I.T.P. SRL-Innovation and Technology Provider (Naples, Italy). The oily formulation was diluted in a solution of ethanol at 10% of DMSO to obtain two different concentrations suitable for in vitro treatments: 50 µg/mL and 70 µg/mL [54,55]. Non-treated cultured macrophages were maintained in incubation media for the relative treatment duration with or without the three different vehicles—sterile water, ethanol, and ethanol—at 10% of DMSO. The final concentration of DMSO in each well was not higher than 0.01%.

### 2.4. Protein Isolation and Western Blotting

Proteins were extracted from control, treated, and non-treated macrophage cultures using radio-immunoprecipitation assay (RIPA) lysis buffer (Millipore, Burlington, MA, USA), following the manufacturer’s instructions. Specific proteins, particularly macrophage surface markers and iron transporters, were detected in total lysates from cell cultures via Western blotting. Membranes were incubated overnight at 4 °C with the following antibodies: anti-CCR7 antibody (1:500, Rabbit. Elabscience, Houston, TX, USA) (molecular weight: 48 KDa), anti-CD86 (1:500, Rabbit, Elabscience, TX, USA) (molecular weight: 70 KDa), anti-CD206 (1:200, Mouse, Santa Cruz Biotechnology, Dallas, TX, USA) (molecular weight: 170 KDa), anti-pSTAT6 antibody (1:500, Rabbit. Elabscience, TX, USA) (molecular weight: 110 KDa), anti-iNOS (1:1000, Rabbit, Sigma Aldrich, St. Louis, MO, USA) (molecular weight: 110 KDa), anti-FPN-1 antibody (1:1000, Rabbit, Novus Biologicals, Milano, Italy) (molecular weight: 62.5 KDa), anti-TfR1 antibody (1:1000, Rabbit, Abcam, Cambridge, UK) (molecular weight: 90 KDa), anti-DMT1 (1:100, Mouse, Santa Cruz Biotechnology, TX, USA) (molecular weight: 64 KDa). Reactive bands were detected using chemiluminescence (Clarity max Western ECL Substrate, Biorad, Hercules, CA, USA) on CHEMIDOC Bio-Rad (BioRad, Hercules, CA, USA). A mouse monoclonal anti β-Actin antibody (1:500, Santa Cruz Biotechnologies, TX, USA) (molecular weight: 43 KDa) was used to check for comparable protein loading and as a housekeeping protein. Images were captured, stored, and analyzed using “Image Lab.Ink 6.1” software.

### 2.5. ELISA

Several ELISA assays were performed to determine the IL-6, IL-4, IL-10, TNF-α, IFN-γ, and Hepcidin concentrations in the cell culture supernatants by using commercially available Human ELISA Kits (Invitrogen by Thermo Fisher, Waltham, MA, USA) according to the manufacturer’s instructions. Briefly, the microplates were coated with monoclonal antibodies specific to cytokines. Standards and supernatants were pipetted into the wells of the microplate and were run in duplicate. After this step, the plate was washed, and enzyme-linked polyclonal antibodies specific for IL-4, IL-10, IL-6, and IFN-γ were added to the wells. The reaction was revealed by the addition of the substrate solution. The optical density was measured at a wavelength of 450 nm using the DAS Italy plate reader (DAS Italy, Palombara Sabina, Italy). Cytokine concentrations (pg/mL) were determined against a standard concentration curve.

### 2.6. Iron Assay

After 24 h of exposure to nutraceutical treatments, cell culture supernatants were collected to measure iron (III). The assay was performed by using the iron assay kit (Abcam, Cambridge, UK) according to the manufacturer’s instructions. Briefly, standard and macrophage supernatants were pipetted into the wells and incubated with an acidic buffer to measure iron released from macrophages in the supernatants. Then, an iron probe was added at 25 °C for 60 min, avoiding direct exposure to light. Released iron reacted with the chromogen, resulting in a colorimetric (593 nm) product proportional to the iron amount. The optical density was measured at a wavelength of 593 nm using the DAS Italy plate reader (DAS Italy, Palombara Sabina, Italy). The iron (II) and total iron (II + III) contents of the test samples (nmol/μL) were determined against a standard concentration curve. The iron (III) content can be calculated as: Iron (III) = Total Iron (II + III) − Iron (II).

### 2.7. MTT Cell Proliferation and Cytotoxicity Assay

Using the colorimetric MTT assay, we assessed the viability and the proliferation of CCS macrophages after 24 h of treatment according to the manufacturer’s instructions (Elabscience, TX, USA). MTT is 3-(4,5-dimethylthiazol-2-yl)-2,5-diphenyl tetrazolium bromide, which can be reduced by some dehydrogenases in mitochondria, forming a crystalline purple product formazan. Formazan can be dissolved by DMSO, and its absorbance is near the 570 nm wavelength. The faster the cells proliferate, the darker the color is, while the more cytotoxic they are, the lighter the color is. There is a linear relationship between the depth of darkness of the color and the number of cells.

### 2.8. Statistical Analysis

Statistical analyses on all the performed experiments were executed using Student’s *t* test to evaluate differences between quantitative variables. We utilized GraphPad Prism version 8.4.2. Each experiment was performed on samples derived from three different subjects, and data are expressed as mean ± SD. A *p* value ≤ 0.05 was considered statistically significant.

## 3. Results

### 3.1. Characterization of Macrophages Derived from CCS Patients

**Figure 1:**
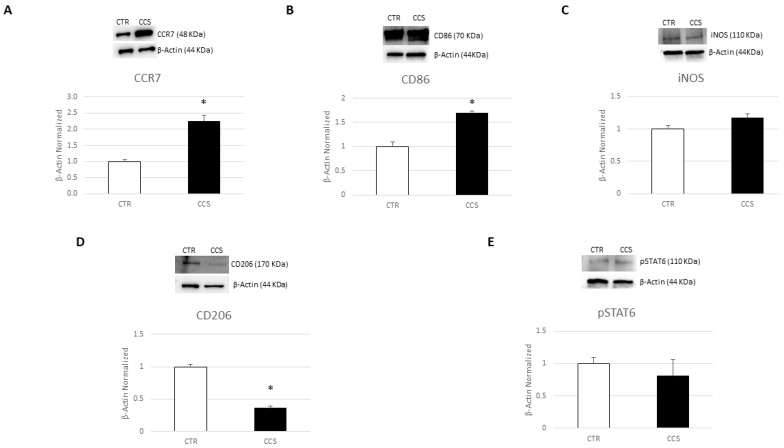
Characterization of macrophages derived from CCS patients. CCR7 (**A**), CD86 (**B**), iNOS (**C**), CD206 (**D**), and pSTAT6 (**E**) protein expression levels in childhood cancer survivors’ (CCS) macrophages compared to those of healthy donors (CTR), evaluated by Western blotting starting from 15 μg of total lysates. The most representative images are displayed. The protein bands were detected through Image Lab Ink 6.1 software “BIORAD”, and the intensity ratios of immunoblots compared to CTR, taken as 1, were quantified after normalizing with the respective controls. The relative quantification for these proteins, normalized for the housekeeping protein β-actin, is represented in the histograms as the mean ± SD. Student’s *t*-test was used for statistical analysis. *, *p* ≤ 0.05 compared to CTR. The uncropped blots are shown in File S1 and Figure S1.

We performed several Western blotting analyses to evaluate protein expression levels for markers linked to M1 and M2 phenotypes in macrophages obtained from the peripheral blood of CCS patients. Biochemical analysis revealed that CCS showed markedly higher levels of CCR7 and CD86, two M1 phenotype markers, compared to healthy donors (CTR) (Figure 1A,B; Supplementary Figure S1A,B). There was also a trend of increasing protein expression levels of another M1 marker, iNOS, although not in a statistically significant manner (Figure 1C; Supplementary Figure S1C). Moreover, we observed a reduction in two different M2 phenotype markers, CD206 and pSTAT6, in CCS macrophages compared to CTR ones (Figure 1D,E; Supplementary Figure S1D,E). These results indicate that, in CCS patients, there is a prevalence of the M1 macrophage phenotype, therefore confirming the alteration of the inflammatory state in CCS patients.

### 3.2. Inflammatory Profiles in Macrophages Derived from CCS Patients

**Figure 2:**
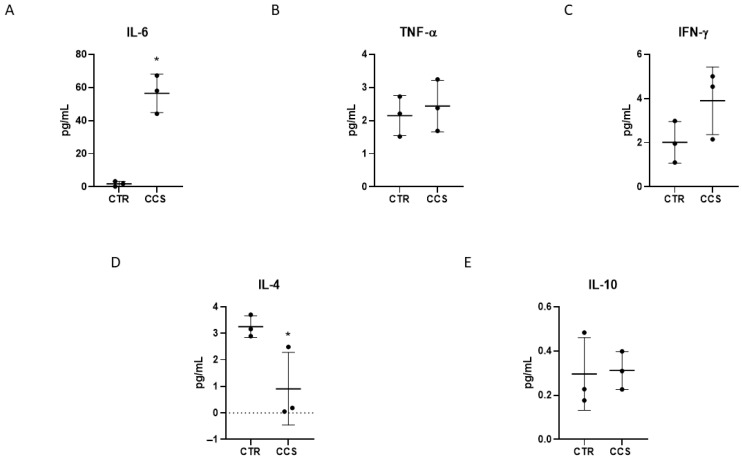
Inflammatory profiles in macrophages derived from CCS patients. Evaluation of IL-6 (**A**), TNF-α (**B**), IFN-γ (**C**), IL-4 (**D**), and IL-10 (**E**) release from childhood cancer survivors’ (CCS) macrophages compared to healthy donors’ (CTR) macrophages, revealed through an enzyme-linked immunosorbent assay (ELISA). The graphs show interleukin levels (pg/mL) as the mean ± standard deviation (SD). Student’s *t*-test was used for statistical analysis. *, *p* ≤ 0.05 compared to CTR.

Enzyme-linked immunosorbent assays (ELISAs), performed to analyze the cytokine release by CCS macrophages compared to CTR, displayed a significant increase in the release of the pro-inflammatory cytokine IL-6, together with a trend of increasing IFN-γ (Figure 2A,C); instead, we noticed slightly increased levels of TNF-α between CCS and CTR macrophages (Figure 2B). Evaluation of the levels of anti-inflammatory cytokines revealed a strong reduction in IL-4 release, but no such variation in IL-10 levels (Figure 2D,E). These ELISA results confirm, once again, the alteration of the inflammatory state with a prevalence of the pro-inflammatory profile.

### 3.3. Iron Metabolism in Macrophages Obtained from CCS Patients

**Figure 3:**
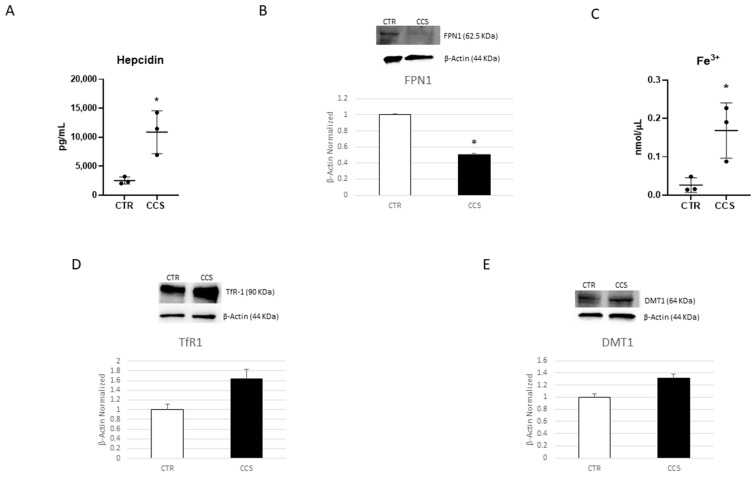
Iron metabolism in macrophages obtained from CCS patients. Evaluation of hepcidin (**A**) release from childhood cancer survivors’ (CCS) macrophages compared to healthy donors’ (CTR) macrophages, revealed through an enzyme-linked immunosorbent assay (ELISA). The graphs show hepcidin levels (pg/mL) as the mean ± standard deviation (SD). Student’s *t*-test was used for statistical analysis. FPN1 (**B**), TfR1 (**D**), and DMT1 (**E**) protein expression levels in CCS patients’ macrophages compared to CTR, evaluated by Western blotting, starting from 15 μg of total lysates. The most representative images are displayed. The protein bands were detected through Image Lab Ink 6.1 software “BIORAD”, the intensity ratios of immunoblots compared to CTR, taken as 1, were quantified after normalizing with the respective controls. The relative quantification for these proteins, normalized for the housekeeping protein β-Actin, is represented in the histograms as the mean ± SD. Student’s *t*-test was used for statistical analysis. Fe^3+^ (**C**) intracellular concentrations (nmol/µL) (**B**) in CTR and CCS patients’ macrophages, determined by iron assay. Histogram shows Fe^3+^ concentration as the mean ± SD. Student’s *t*-test was used for statistical analysis. * *p* ≤ 0.05 compared to CTR. The uncropped blots are shown in File S1 and Figure S2.

Iron metabolism in CCS macrophages was assessed by evaluating hepcidin levels, intracellular iron concentration, and FPN-1 protein expression. Interestingly, we revealed elevated levels of hepcidin in CCS macrophages compared to CTR ones (Figure 3A; Supplementary Figure S2A). This result is in accordance with the increase in IL-6 release observed in CCS macrophages (Figure 2A). As a consequence of the increment in hepcidin levels, we observed not only a statistical reduction in FPN-1, but also an increase in [Fe^3+^] in CCS macrophages compared to CTR (Figure 3B,C), thus confirming the close correlation between iron metabolism and inflammation. Moreover, we also observed an increase in both the iron importers, TfR1 and DMT1, albeit not in statistically significant manner (Figure 3D,E, Supplementary Figure S2B,C).

### 3.4. Effect of Resveratrol, Curcumin, and Oil-Enriched Lycopene on Macrophage Proliferation

**Figure 4:**
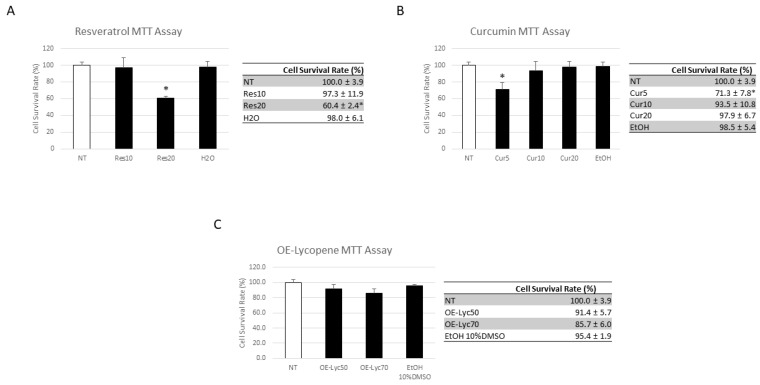
Effects of resveratrol, curcumin, and oil-enriched lycopene on proliferation of CCS macrophages and evaluation of its cytotoxicity. Cell survival rate in childhood cancer survivors (CCS) patients’ macrophages after 24 h of treatment with resveratrol (Res) at different concentrations (10 µM and 20 µM) and its vehicle (sterile water—H_2_O) (**A**); curcumin (Cur) at different concentrations (5 µM, 10 µM, 20 µM) and its vehicle (ethanol—EtOH) (**B**); and oil-enriched-lycopene (OE-Lyc) at different concentrations (50 µg/mL and 70 µg/mL) and its vehicle (EtOH at 10% of DMSO) (**C**). The results are presented as the mean percentage ± standard deviation percentage (SD). Student’s *t*-test was used for statistical analysis. * *p* ≤ 0.05 compared to non-treated (NT) macrophages.

We performed an MTT assay to evaluate the effects of Res, Cur, and OE-Lyc on the proliferation of CCS macrophages and the cytotoxicity of these nutraceuticals. Cur and OE-Lyc did not significantly alter the proliferation percentage of CCS macrophages compared to that of the non-treated population (NT) (Figure 4B,C; Supplementary Figure S3B,C). Although a reduction in the cell proliferation after the treatment with Cur5 could be observed, 70% viable cells are not an index of the drug cytotoxicity. Therefore, these results suggest that Cur and OE-Lyc are not cytotoxic to CCS macrophages. Considering Res treatment, we observed a reduction in CCS macrophage proliferation rate after treatment with Res (20 µM) (Figure 4A; Supplementary Figure S3A).

### 3.5. Effect of Resveratrol, Curcumin, and Oil-Enriched Lycopene on Macrophage Phenotype

We evaluated the effects of Res, Cur, and OE-Lyc on macrophage polarization markers by performing Western blotting.

**Figure 5:**
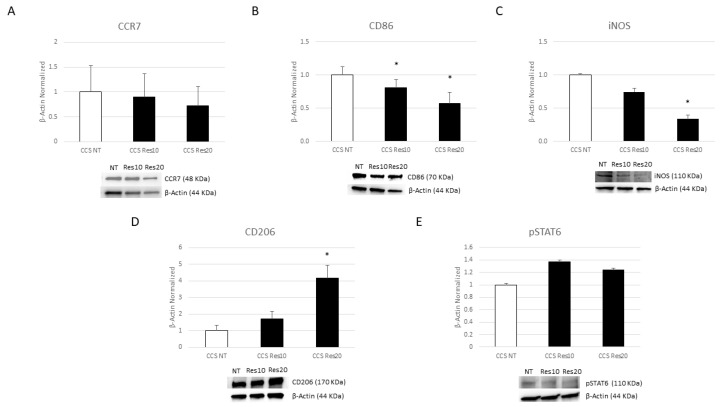
Effects of resveratrol on macrophage phenotype. CCR7 (**A**), CD86 (**B**), iNOS (**C**), CD206 (**D**), and pSTAT6 (**E**) protein expression levels in childhood cancer survivors’ (CCS) macrophages after 24 h treatment with resveratrol (Res) at different concentrations (10 µM and 20 µM), evaluated by Western blotting, starting from 15 μg of total lysates. The most representative images are displayed. The protein bands were detected through Image Lab Ink 6.1 software “BIORAD”. The intensity ratios of immunoblots compared to non-treated (NT) macrophages, taken as 1, were quantified after normalizing with the respective controls. The relative quantification for these proteins, normalized for the housekeeping protein β-Actin, is represented in the histograms as the mean ± SD. Student’s *t*-test was used for statistical analysis. *, *p* ≤ 0.05 compared to NT. The uncropped blots are shown in File S1.

After Res treatment, we interestingly observed a statistically significant reduction in the M1 marker CD86, which was more marked at a concentration of 20 µM compared to the untreated samples (CCS NT) (Figure 5B). We also revealed a decreasing trend of both CCR7 and iNOS, which was statistically significant at a concentration of 20 µM compared to NT macrophages (Figure 5A,C). Moreover, Res was also able to significantly increase the protein expression levels of the M2 phenotype marker CD206, and this result was more evident after Res 20 µM treatment (Figure 5D). We also reported an elevated level of pSTAT6 expression after Res treatment at a concentration of 10 µM, but not in a statistically significant manner (Figure 5E).

**Figure 6:**
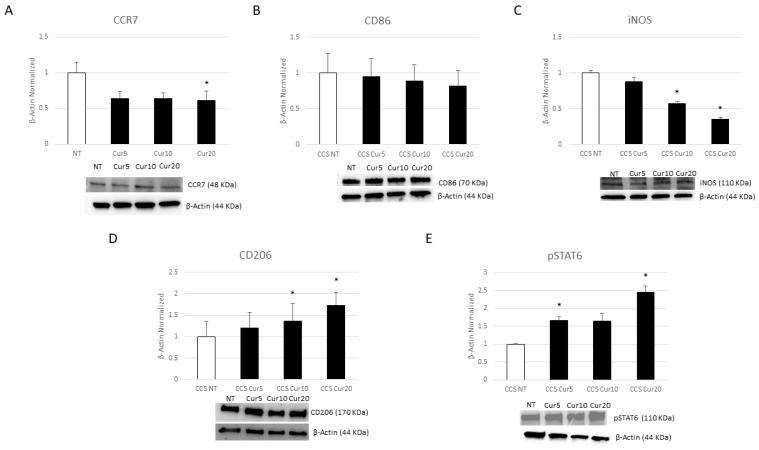
Effects of curcumin on macrophage phenotype. CCR7 (**A**), CD86 (**B**), iNOS (**C**), CD206 (**D**), and pSTAT6 (**E**) protein expression levels in childhood cancer survivors’ (CCS) macrophages after 24 h treatment with curcumin (Cur) at different concentrations (5 µM, 10 µM, and 20 µM), evaluated by Western blotting, starting from 15 μg of total lysates. The most representative images are displayed. The protein bands were detected through Image Lab Ink 6.1 software “BIORAD”, and the intensity ratios of immunoblots compared to non-treated (NT) macrophages, taken as 1, were quantified after normalizing with the respective controls. The relative quantification for these proteins, normalized for the housekeeping protein β-Actin, is represented in the histograms as the mean ± SD. Student’s *t*-test was used for statistical analysis. *, *p* ≤ 0.05 compared to NT. The uncropped blots are shown in File S1.

After Cur administration, we observed a statistically significant decrease in iNOS protein expression levels at 10 µM and 20 µM (Figure 6C), and a trend of a reduction in CCR7, which was statistically significant at 20 µM. We observed a similar trend for CD86 protein expression levels compared to NT macrophages as well, even though it was not statistically significant (Figure 6A,B). Treatment with Cur (20 µM) induced a strong increase in both M2 phenotype markers, CD206 and pSTAT6, compared to NT cells (Figure 6D,E).

**Figure 7:**
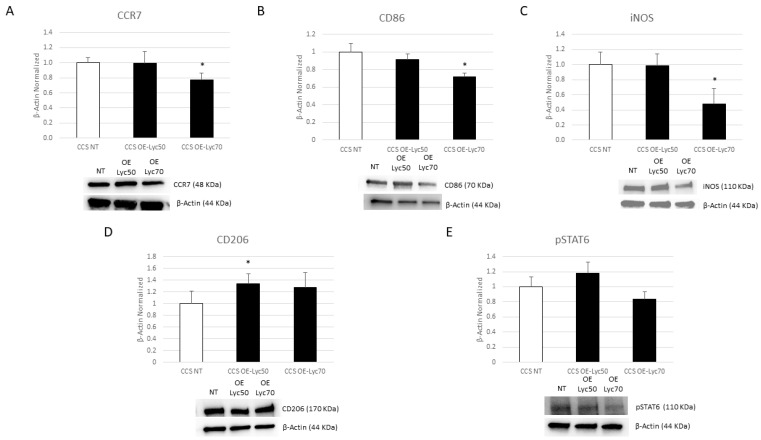
Effects of oil-enriched lycopene on macrophage phenotype. CCR7 (**A**), CD86 (**B**), iNOS (**C**), CD206 (**D**), and pSTAT6 (**E**) protein expression levels in childhood cancer survivors’ (CCS) macrophages after 24 h treatment with oil-enriched lycopene (OE-Lyc) at different concentrations (50 µg/mL and 70 µg/mL), evaluated by Western blotting, starting from 15 μg of total lysates. The most representative images are displayed. The protein bands were detected through Image Lab Ink 6.1 software “BIORAD”, and the intensity ratios of immunoblots compared to non-treated (NT) macrophages, taken as 1, were quantified after normalizing with the respective controls. The relative quantification for these proteins, normalized for the housekeeping protein β-actin, is represented in the histograms as the mean ± SD. Student’s *t*-test was used for statistical analysis. *, *p* ≤ 0.05 compared to NT. The uncropped blots are shown in File S1.

OE-Lyc (70 µg/mL) treatment induced a strong reduction in CD86 and iNOS protein expression and determined a reduction in CCR7 expression levels in a statistically significant manner (Figure 7A–C). Moreover, we also observed an increasing trend of the M2 phenotype marker CD206 after OE-Lyc treatment, as well as a reduction in pSTAT6 compared to NT cells (Figure 7D,E). Although some results were not statistically significant, the collected data let us suppose that Res, Cur, and Lyc could determine a phenotype switch from the pro-inflammatory M1 macrophage phenotype towards the anti-inflammatory M2 one.

### 3.6. Effect of Resveratrol, Curcumin, and Oil-Enriched Lycopene on Inflammatory State

**Figure 8:**
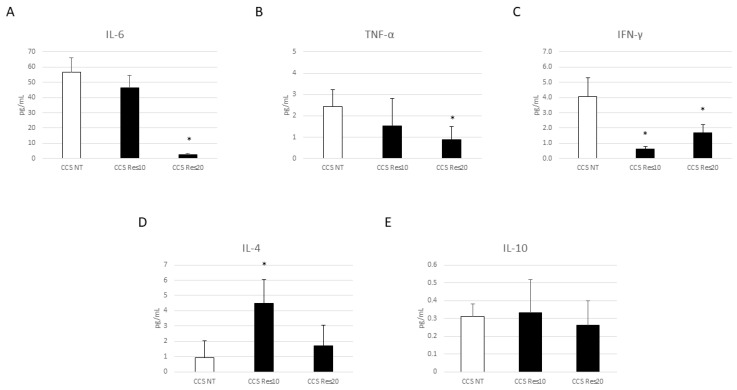
Effect of resveratrol on inflammatory state. Evaluation of IL-6 (**A**), TNF-α (**B**), IFN-γ (**C**), IL-4 (**D**), and IL-10 (**E**) release from childhood cancer survivors’ (CCS) macrophages after 24 h treatment with resveratrol (Res) at different concentrations (10 µM and 20 µM), revealed through an enzyme-linked immunosorbent assay (ELISA). The graphs show interleukin levels (pg/mL) as the mean ± standard deviation (SD). Student’s *t*-test was used for statistical analysis. *, *p* ≤ 0.05 compared to NT.

To evaluate the effects of Res, Cur, and OE-Lyc on the inflammatory state, we performed several ELISA tests. We observed a strong reduction in the pro-inflammatory cytokines IL-6, TNF-α, and IFN-γ after Res (20 µM) administration in CCS macrophages (Figure 8A–C); moreover, we also observed a statistically significant increase in IL-4 release after Res (10 µM) treatment (Figure 8D). We did not observe any variation in IL-10 release after Res administration (Figure 8E).

**Figure 9:**
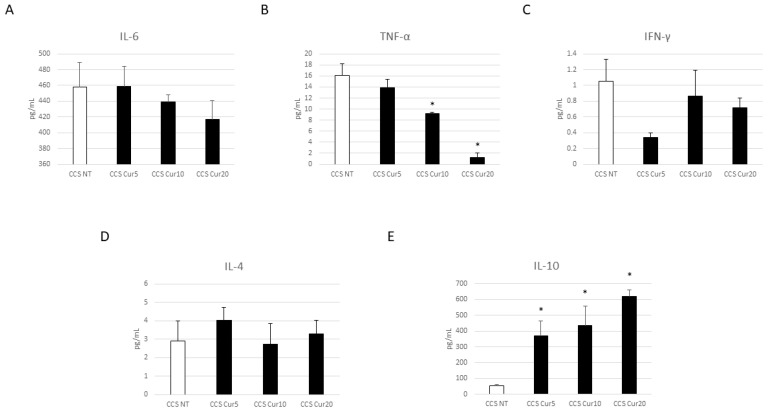
Effect of curcumin on inflammatory state. Evaluation of IL-6 (**A**), TNF-α (**B**), IFN-γ (**C**), IL-4 (**D**), and IL-10 (**E**) release from childhood cancer survivors’ (CCS) macrophages after 24 h treatment with curcumin (Cur) at different concentrations (5 µM, 10 µM, and 20 µM), revealed through an enzyme-linked immunosorbent assay (ELISA). The graphs show interleukin levels (pg/mL) as the mean ± standard deviation (SD). Student’s *t*-test was used for statistical analysis. *, *p* ≤ 0.05 compared to NT.

**Figure 10:**
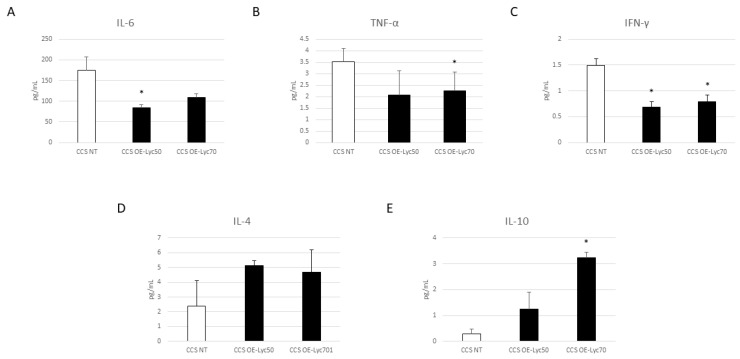
Effect of oil-enriched lycopene on inflammatory state. Evaluation of IL-6 (**A**), TNF-α (**B**), IFN-γ (**C**), IL-4 (**D**), and IL-10 (**E**) release from childhood cancer survivors’ (CCS) macrophages after 24 h treatment with oil-enriched lycopene (OE-Lyc) at different concentrations (50 µg/mL and 70 µg/mL), revealed through an enzyme-linked immunosorbent assay (ELISA). The graphs show interleukin levels (pg/mL) as the mean ± standard deviation (SD). Student’s *t*-test was used for statistical analysis. *, *p* ≤ 0.05 compared to NT.

Interestingly, Cur and OE-Lyc treatments induced a significant reduction in both pro-inflammatory cytokines, TNF-α and IFN-γ (Figure 9B,C and Figure 10B,C). Considering the effect on IL-6 levels, we observed an extraordinarily strong reduction in its release after OE-Lyc (50 µg/mL) administration (Figure 10A); Cur induced a decreasing trend of IL-6 release, but not in a statistically significant manner (Figure 9A). Moreover, both Cur and OE-Lyc administration induced an increase in IL-4 (Figure 9D and Figure 10D) and a statistical increase in IL-10 release (Figure 9E and Figure 10E), thus supporting the hypothesis that both drugs could have anti-inflammatory activity not only by containing the release of pro-inflammatory cytokines, but also by increasing levels of anti-inflammatory cytokines.

### 3.7. Effect of Resveratrol, Curcumin, and Oil-Enriched Lycopene on Iron Metabolism

**Figure 11:**
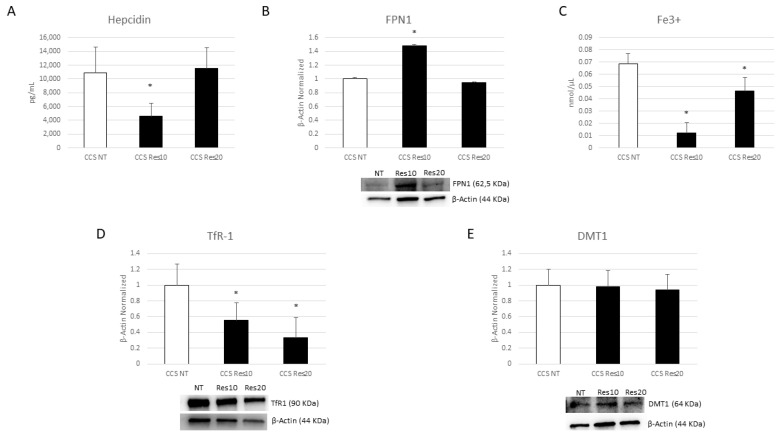
Effect of resveratrol on iron metabolism. Evaluation of hepcidin (**A**) release from childhood cancer survivors’ (CCS) macrophages after 24 h treatment with resveratrol (Res) at different concentrations (10 µM and 20 µM), revealed through an enzyme-linked immunosorbent assay (ELISA). The graphs show hepcidin levels (pg/mL) as the mean ± standard deviation (SD). Student’s *t*-test was used for statistical analysis. FPN1 (**B**), TfR1 (**D**), and DMT1 (**E**) protein expression levels in CCS patients’ macrophages after 24 h treatment with Res at different concentrations (10 µM and 20 µM), evaluated by Western blotting, starting from 15 μg of total lysates. The most representative images are displayed. The protein bands were detected through Image Lab Ink 6.1 software “BIORAD”, and the intensity ratios of immunoblots compared to non-treated (NT) macrophages, taken as 1, were quantified after normalizing with the respective controls. The relative quantification for these proteins, normalized for the housekeeping protein β-Actin, is represented in the histograms as the mean ± SD. Student’s *t*-test was used for statistical analysis. Fe^3+^ intracellular concentrations (nmol/µL) (**C**) in CCS patients’ macrophages after 24 h treatment with Res at different concentrations (10 µM and 20 µM), determined by iron assay. Histogram shows Fe^3+^ concentration as the mean ± SD. Student’s *t*-test was used for statistical analysis. * *p* ≤ 0.05 compared to NT. The uncropped blots are shown in File S1.

To study the effects of nutraceuticals on iron metabolism in macrophages obtained from CCS patients, we firstly evaluated the hepcidin-FPN-1 axis. We revealed a statistically significant reduction in hepcidin production after Res (10 µM) administration (Figure 11A). This observed decrease interestingly corresponds with the increase in FPN-1 expression levels and the strong reduction in intracellular iron concentration (Figure 11B,C), suggesting that Res exerts its anti-inflammatory properties by modulating the IL-6-hepcidin-FPN-1 axis.

**Figure 12:**
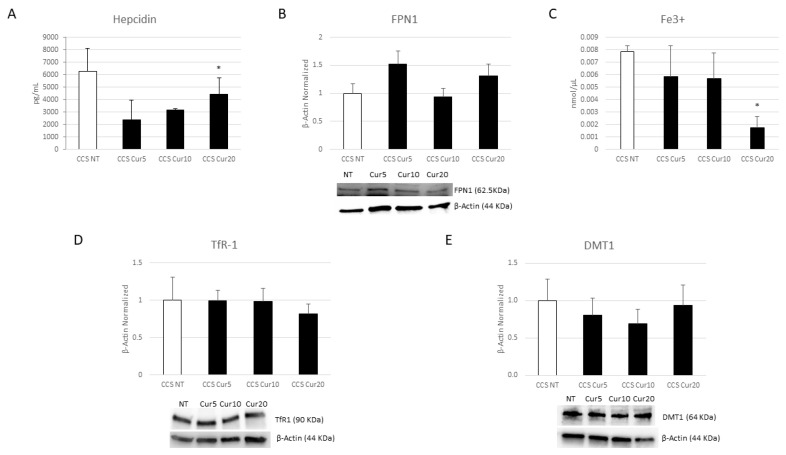
Effect of Curcumin on iron metabolism. Evaluation of Hepcidin (**A**) release from childhood cancer survivors (CCS) patients’ macrophages after 24-h treatment with Curcumin (Cur) at different concentrations (5 µM, 10 µM, and 20 µM) revealed through an enzyme-linked immunosorbent assay (ELISA). The graphs show hepcidin levels [pg/mL] as the mean ± standard deviation (SD). Student’s *t*-test has been used for statistical analysis. FPN1 (**B**), TfR1 (**D**), and DMT1 (**E**) protein expression levels in CCS patients’ macrophages after 24-h treatment with Cur at different concentrations (5 µM, 10 µM, and 20 µM) evaluated by Western Blot, starting from 15 μg of total lysates. The most representative images are displayed. The protein bands were detected through Image Lab. Ink 6.1 software “BIORAD”, and the intensity ratios of immunoblots compared to non-treated (NT) macrophages, taken as 1, were quantified after normalizing with respective controls. The relative quantification for these proteins, normalized for the housekeeping protein β-Actin, is represented in the histograms as the mean ± SD. Student’s *t*-test has been used for statistical analysis. Fe^3+^ intracellular concentrations (nmol/µL) (**C**) in CCS patients’ macrophages after 24-h treatment with Cur at different concentrations (5 µM, 10 µM, and 20 µM), determined by iron assay. Histogram shows Fe^3+^ concentration as the mean ± SD. Student’s *t*-test has been used for statistical analysis. * *p* ≤ 0.05 compared to NT. The uncropped blots are shown in File S1.

**Figure 13:**
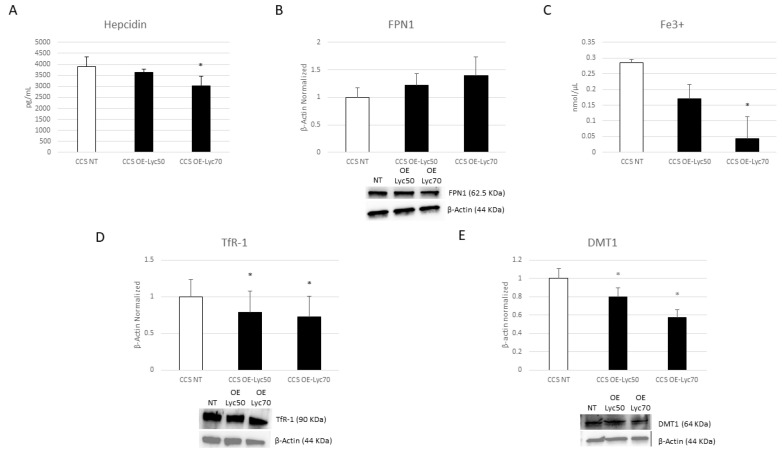
Effect of oil-enriched lycopene on iron metabolism. Evaluation of hepcidin (**A**) release from childhood cancer survivors’ (CCS) macrophages after 24 h treatment with oil-enriched lycopene (OE-Lyc) at different concentrations (50 µg/mL and 70 µg/mL), revealed through an enzyme-linked immunosorbent assay (ELISA). The graphs show hepcidin levels (pg/mL) as the mean ± standard deviation (SD). Student’s *t*-test was used for statistical analysis. FPN1 (**B**), TfR1 (**D**), and DMT1 (**E**) protein expression levels in CCS patients’ macrophages after 24 h treatment with OE-Lyc at different concentrations (50 µg/mL and 70 µg/mL), evaluated by Western blotting, starting from 15 μg of total lysates. The most representative images are displayed. The protein bands were detected through Image Lab Ink 6.1 software “BIORAD”, and the intensity ratios of immunoblots compared to non-treated (NT) macrophages, taken as 1, were quantified after normalizing with the respective controls. The relative quantification for these proteins, normalized for the housekeeping protein β-actin, is represented in the histograms as the mean ± SD. Student’s *t*-test was used for statistical analysis. Fe^3+^ intracellular concentrations (nmol/µL) (**C**) in CCS patients’ macrophages after 24 h treatment with OE-Lyc at different concentrations (50 µg/mL and 70 µg/mL), determined by iron assay. Histogram shows Fe^3+^ concentration as the mean ± SD. Student’s *t*-test was used for statistical analysis. * *p* ≤ 0.05 compared to NT. The uncropped blots are shown in File S1.

We demonstrated that Cur is also able to modulate iron metabolism, observing a trend to increase of FPN1 expression levels after Cur treatment (Figure 12B) and statistically significant results after Cur [20 µM] administration, which induced a strong reduction in both hepcidin and intracellular iron levels (Figure 12A,C). The same results were also obtained with OE-Lyc [70 µg/mL], with a marked effect on intracellular iron concentration (Figure 13A–C).

Subsequently, we also evaluated the effects of these compounds on two important iron importers, TfR1 and DMT1. Res is able to reduce TfR1 protein expression levels in an incredibly significant manner, but it has no statistical effect on DMT1 expression (Figure 11D,E). We also observed a reduction after the Cur and OE-Lyc treatments. Cur induced decreases in both DMT1 and TfR1 (Figure 12D,E). For OE-Lyc, we observed statistically significant reductions in both TfR1 and DMT1 protein expression levels after OE-Lyc (50 µg/mL) and OE-Lyc (70 µg/mL) administration (Figure 13D,E).

All these results let us suggest, for the first time, that these nutraceuticals exert anti-inflammatory properties through novel mechanisms of action, by modulating iron metabolism.

## 4. Discussion

The increasing rate of CCS is an important achievement in pediatric oncology due to advancements in the available anti-cancer treatments; nevertheless, they exhibit long-term side effects. [1,2,56,57]. These anti-cancer treatments lead to the onset of late morbidity, which interferes with normal biological processes, specifically aging. This ultimately leads to frailty, causing early exposure to the risk of prematurely developing age-related chronic diseases, frequent hospitalizations, and—in severe cases—early mortality during adulthood [2,6,7,8].

Treatments like chemotherapy and radiotherapy are known to cause a low-grade chronic inflammatory state followed by an accelerated “aging” process—called “inflamm-aging” [2,58]. These treatments cause the production of compounds like RNS and ROS, which play a role in inflammation by determining DNA damage, pro-inflammatory cytokine production, and activation and proliferation of lymphocytes and classically activated macrophages (M1) [2,11,12]. Hence, the recognition of inoffensive therapeutical approaches able to counteract related consequences of inflammation as well as itself could be useful.

M1 and M2 are two activated phenotypes of macrophages [13,14]. The M1 phenotype usually exhibits pro-inflammatory, anti-cancer, and antimicrobial functions, whereas M2 macrophages exert anti-inflammatory and immunosuppressive effects [13,14]. There is a pivotal role of macrophages in iron homeostasis as they participate in in iron efflux: M1 macrophages are responsible for iron internalization; in contrast, the M2 phenotype shows its involvement in iron release [13,16,17,18]. Iron plays a crucial role in mammalian cells by participating in several biological processes, and cellular damage is correlated with alteration of its metabolism [18,59]. A close correlation between iron metabolism and inflammation has already been determined. Indeed, during inflammation, elevated levels of IL-6 determine an increase in the production and activation of hepcidin, which binds FPN-1 and causes its degradation, resulting in an increase in intracellular iron concentration [16,22]. Iron accumulation in macrophages alters their activity, contributing to the exacerbation of inflammation [13,14,22]. Therefore, macrophages, iron, and its metabolism could be proposed as novel and interesting therapeutic targets to counteract inflammation, which is a common condition in CCS. Nutraceuticals are natural compounds with noteworthy emerging antioxidant, anti-inflammatory, and anti-cancer properties [23,25,27,28,29,60]. Res, Cur, and Lyc are nutraceuticals already known for their therapeutic properties [32,39,41,42,43,44,45,46,47,61,62]. They show anti-inflammatory and antioxidant activities by modulating the release and the production of several pro-inflammatory cytokines and mediators of ROS production in vitro and in vivo [34,35,36,37,39,41,42,43,44,45,46,47,48,49,50,63]. In particular, Res exerts its anti-inflammatory, antioxidant, and anti-aging properties by inhibiting IFN-γ and IL-2 release by lymphocytes and TNF-α or IL-12 production by macrophages [34]. It has been demonstrated that Res shows anti-inflammatory effects in in vitro macrophages by reducing the expression level of iNOS as well as the release of the pro-inflammatory cytokines IL-1β, IL-6, and TNF-α [64,65,66,67,68,69,70]. Interestingly, these results were also confirmed in U-937 and RAW 264.7 macrophages; indeed, Res treatment reduces TNF-α release and the gene expression of IL-1β, IL-6, and TNF-α [65]. Jeong et al. demonstrated that Res is also able to induce a macrophage switch from the M1 pro-inflammatory profile towards the M2 anti-inflammatory type in high-fat-diet mice, counteracting inflammation, which is a typical condition in obesity [71]. It has been reported that Res is also able to reduce IL-17 release in vitro, thus reducing the inflammatory status [66]. The anti-inflammatory activity of Res also occurs due to the inhibition of ROS, NO, and iNOS production, counteracting the oxidative stress and the consequent impairment of inflammatory responses typical of different disorders, like cancer and chronic inflammation [32,72]. Also, Cur shows anti-inflammatory effects by reducing the synthesis of pro-inflammatory compounds such as IL-6, IL-1β, IL-1, and TNF-α. It can also contain oxidative stress, reducing the production of iNOS and NO [39,41,42,43,44,45,46]. Interestingly, Gao et al. demonstrated that Cur determines a macrophage switch from the pro-inflammatory phenotype M1 towards the anti-inflammatory phenotype M2 [61,62]. Lyc is another nutraceutical known to have anti-inflammatory and antioxidant activities [47,48,49,50]. Feng et al. demonstrated that Lyc counteracts inflammation in LPS-activated RAW264.7 macrophages by inhibiting MAPK and NF-kB pathways and obstructing NO formation and IL-6 release [63]. It has also been reported that Lyc is able to regulate macrophage polarization by causing a significant reduction in M1 macrophages/Kupffer cells in the liver [73].

Considering the already known properties of these compounds, we decided to investigate innovative mechanisms of action mediating the anti-inflammatory effects of Res, Cur, and OE-Lyc in macrophages obtained from CCS patients.

Firstly, we dissected the inflammatory condition of CCS patients by focusing on evaluation of their macrophage populations. We demonstrated that, in CCS patients, there is a prevalence of the M1 pro-inflammatory macrophage phenotype, thus both confirming the existence of a low-grade chronic inflammatory state and suggesting the contribution of M1 macrophages to this impaired inflammatory condition. Indeed, the increased levels of CCR7 and CD86 proteins together with the decreased levels of pSTAT6 and CD206 protein expression prompt us to propose the hypothesis that the low-grade chronic inflammatory state could be a consequence of an excess of M1 macrophages. Our research confirmed the existence of the well-known low-grade chronic inflammation in CCS patients by observing prominent levels of pro-inflammatory cytokines IL-6 and IFN-γ together with a strong decrease in the anti-inflammatory cytokine IL-4 in CCS macrophages compared to CTR ones. Interestingly, we also demonstrated, for the first time, an alteration of iron metabolism in CCS macrophages. Indeed, because of the observed elevated levels of IL-6, we highlighted a strong increase in hepcidin production by CCS macrophages compared to CTR ones, which resulted in a reduction in FPN-1 expression levels and, consequently, in an increase in intracellular iron concentration. Moreover, we also highlighted that CCS macrophages show increased expression of both iron importers, DMT1 and TfR1, which contribute to increasing intracellular iron levels. Considering the close relationship between iron and inflammation, it could be possible that the impairment of iron metabolism is one of the causes of inflammation in CCS. Therefore, targeting iron and its metabolism could be a novel strategy to reduce inflammation and, consequently, to prevent all the related consequences. Certainly, the proposal of new therapeutic interventions that are not properly pharmacological would be a desirable strategy to orderly counteract and contain inflammation—the main cause of the onset of inflamm-aging—and, at the same time, to avoid further pharmacological treatments for CCS patients. Based on this conceptual paradigm, we decided to evaluate the effects of exposure to different nutraceuticals which are already known to have anti-inflammatory and antioxidant activities. In particular, we administered Res, Cur, and OE-Lyc at different concentrations on CCS macrophage cell cultures and evaluated their effects after 24 h of incubation. Firstly, we confirmed their anti-inflammatory properties by observing reductions in three M1 phenotype markers—CCR7, CD86, and iNOS—and concurrent increases in M2 phenotype markers—CD206 and pSTAT6. These results confirmed the already-known capability of Res and Cur to induce a macrophage switch towards the M2 anti-inflammatory phenotype and suggested—for the first time in our knowledge—that OE-Lycopene can also exert this property. Moreover, we also confirmed the anti-inflammatory effects of these compounds by revealing a strong decrease in the pro-inflammatory cytokines IL-6, TNF-α, and IFN-γ, and also by observing a trend of increases in the two anti-inflammatory cytokines IL-4 and IL-10.

We also highlighted the effects of Res, Cur, and OE-Lyc on iron metabolism. Surprisingly, we observed that by administering these three compounds, we could modulate iron metabolism by determining a strong and significant reduction in intracellular iron concentration. In particular, treatments with Res, Cur, and OE-Lyc not only determined a strong reduction in IL-6 release, as already shown, but at the same time induced both a decrease in hepcidin levels and an increase in FPN-1 expression levels, thus resulting in a strong reduction in intracellular iron concentration. These results show both a direct effect of these nutraceuticals on each single component of iron metabolism (IL-6, hepcidin, FPN-1, and iron) and an indirect effect due to modulation of IL-6 levels, which occurs upstream of the IL-6-hepcidin-FPN1 axis.

Even though some results are not statistically significant, considering the trends and general flow of evidence, an overall positive effect of the treatments could be expected. Remarkably, for the first time, we demonstrated that Res, Cur, and OE-Lyc can exert their anti-inflammatory properties by modulating the iron metabolism, therefore underlining a new anti-inflammatory mechanism of action.

## 5. Conclusions

In conclusion, we evaluated the involvement of M1 macrophages and the alteration of iron metabolism in CCS patients impaired by inflammatory conditions for the first time. Overall, we suggest that targeting iron and its metabolism could be a novel therapeutic target to counteract inflammation in CCS patients, highlighting for the first time the capability of nutraceuticals to exert their anti-inflammatory properties by modulating iron metabolism.

The obtained results are very promising and lead to the hypothesis that nutraceuticals administration could be an important and innovative therapeutic strategy to counteract and prevent the low-grade chronic inflammatory state in CCS by preventing the onset of inflamm-aging-related consequences, avoiding aggressive therapeutic strategies which would further complicate the health and quality of life of CCS. Non-pharmacological intervention strategies, targeting inflamm-aging in order to restore or prevent immunological imbalance, are strongly needed to grant long-term health to CCS, to fully provide them with the best and healthiest lifespans with which to take advantage of improvements in oncological therapies. Although our study provides interesting and important findings, there are some limitations that can certainly be overcome: an expansion of the size of our samples and an extension of the follow-up period could be beneficial. Moreover, in the future, further in vitro or in vivo investigations will be needed to validate our findings in order to propose these compounds as alternative therapeutic strategies to counteract inflamm-aging in CCS.

## Data Availability

The data presented in this study are available in this article (and supplementary material).

## Supplementary Materials

The following supporting information can be downloaded at: https://www.mdpi.com/article/10.3390/cancers16040714/s1, Figure S1: Characterization of macrophages derived from CCS patients; Figure S2: Iron metabolism in macrophages obtained from CCS patients; Figure S3: Effects of Resveratrol, Curcumin, and Oil Enriched-Lycopene on proliferation of CCS macrophages and evaluation of its cytotoxicity; File S1: Full pictures of the Western blots.
