# Supplementary material for: Role of Nutraceuticals in Counteracting Inflammation in In Vitro Macrophages Obtained from Childhood Cancer Survivors
Source: Cancers (Basel). 2024 Feb 8;16(4):714. doi: 10.3390/cancers16040714 (PMC10886672; doi:10.3390/cancers16040714)
Supplement: Supplementary file 1 [file cancers-16-00714-s001.zip › Supplementary Figures.pdf]

Supplementary Figure S1

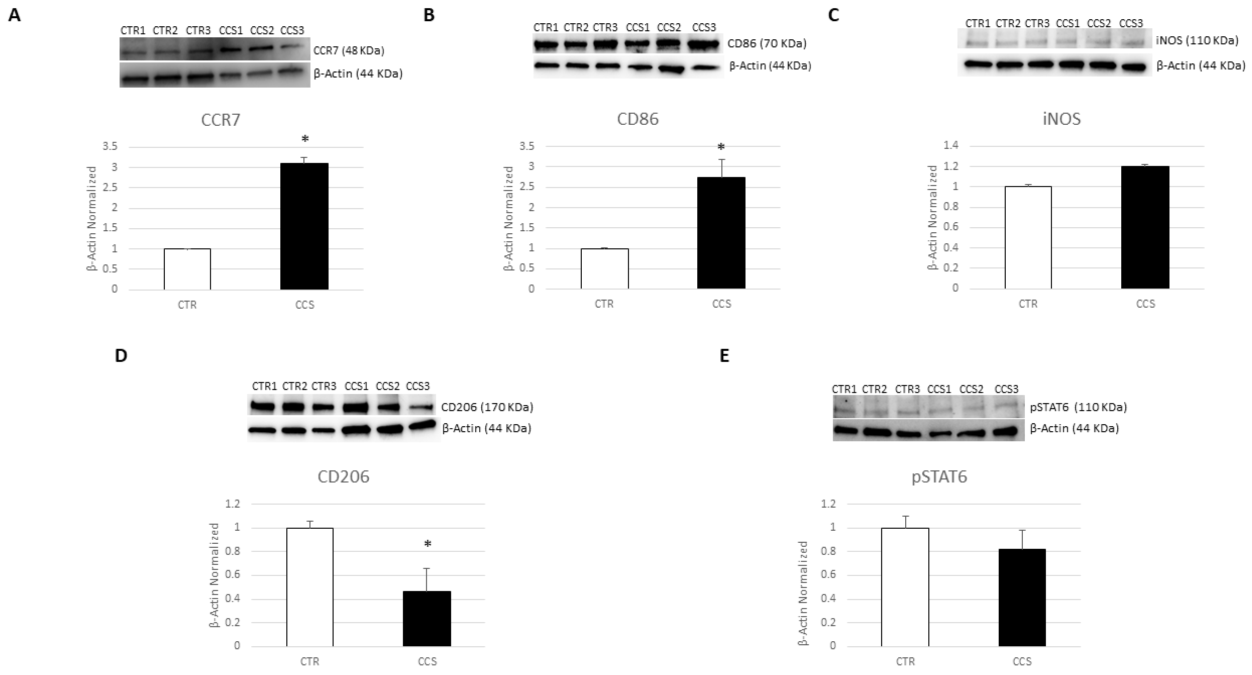

Figure S1. Characterization of macrophages derived from CCS patients. CCR7 (A), CD86 (B), iNOS (C), CD206 (D), and pSTAT6 (E) protein expression levels in 3 childhood cancer survivors (CCS) patients' macrophages compared to 3 healthy donors (CTR) macrophages evaluated by Western Blot, starting from 15 µg of total lysates. The most representative images are displayed. The protein bands were detected through Image Lab. Ink software "BIORAD", and the intensity ratios of immunoblots compared to CTR, taken as 1, were quantified after normalizing with respective controls. The relative quantification for these proteins, normalized for the housekeeping protein β-Actin, is represented in the histograms as the mean ± SD. Students' t-test has been used for statistical analysis. *, $p \le 0.05$ compared to CTR.

Supplementary Figure S2

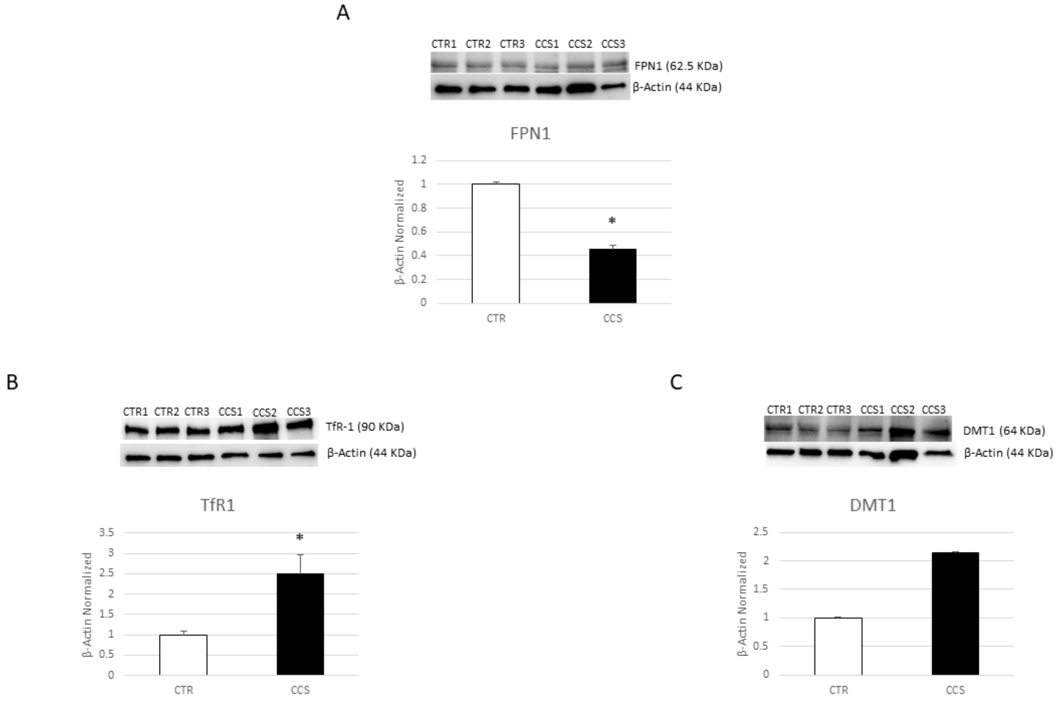

Figure S2. Iron metabolism in macrophages obtained from CCS patients. FPN1 (A), TfR1 (B), and DMT1 (C) protein expression levels in 3 childhood cancer survivors (CCS) patients' macrophages compared to 3 healthy donors (CTR), evaluated by Western Blot, starting from 15 µg of total lysates. The most representative images are displayed. The protein bands were detected through Image Lab. Ink software "BIORAD", and the intensity ratios of immunoblots compared to CTR, taken as 1, were quantified after normalizing with respective controls. The relative quantification for these proteins, normalized for the housekeeping protein β-Actin, is represented in the histograms as the mean ± standard deviation (SD). Students' t-test has been used for statistical analysis.

Supplementary Figure S3

A

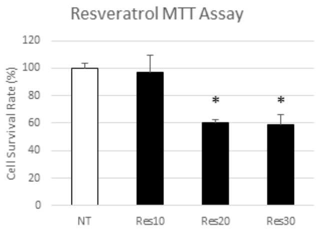

	Cell Survival Rate (%)
NT	100.0 ± 3.9
Res10	97.3 ± 11.9
Res20	60.4 ± 2.4*
Res30	58.7 ± 7.7

B

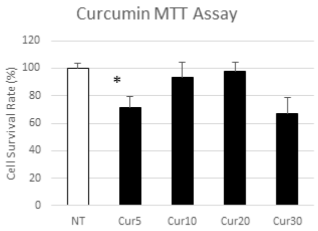

	Cell Survival Rate (%)
NT	100.,0 ± 3.9
Cur5	71.3 ± 7.8*
Cur10	93.5 ± 10.8
Cur20	97.9 ± 6.7
Cur30	67.3 ± 11.4

C

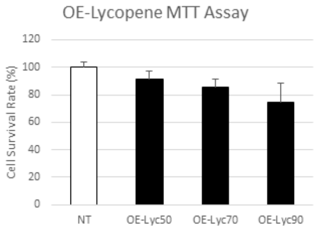

	Cell Survival Rate (%)
NT	100.0 ± 3.9
OE-Lyc50	91.4 ± 5.7
OE-Lyc70	85.7 ± 6.0
OE-Lyc90	74.3 ± 14.3

Figure S3. Effects of Resveratrol, Curcumin, and Oil Enriched-Lycopene on proliferation of CCS macrophages and evaluation of its cytotoxicity. Cell survival rate in childhood cancer survivors (CCS) patients' macrophages after 24 h of treatment with Resveratrol (Res) at different concentrations (10µM, 20µM and 30µM) (**A**), Curcumin (Cur) at different concentrations (5µM, 10µM, 20µM and 30µM) (B), and Oil Enriched-Lycopene (OE-Lyc) at different concentrations (50 µg/mL, 70 µg/mL and 90µg/mL) (C). The results are presented as the mean percentage ± standard deviation percentage (SD). A Student's t-test has been used for statistical analysis. *$p \leq 0.05$ compared to non-treated (NT) macrophages.
