# Supplementary figures and images for: Role of Nutraceuticals in Counteracting Inflammation in In Vitro Macrophages Obtained from Childhood Cancer Survivors
Source: Cancers (Basel). 2024 Feb 8;16(4):714. doi: 10.3390/cancers16040714 (PMC10886672; doi:10.3390/cancers16040714)

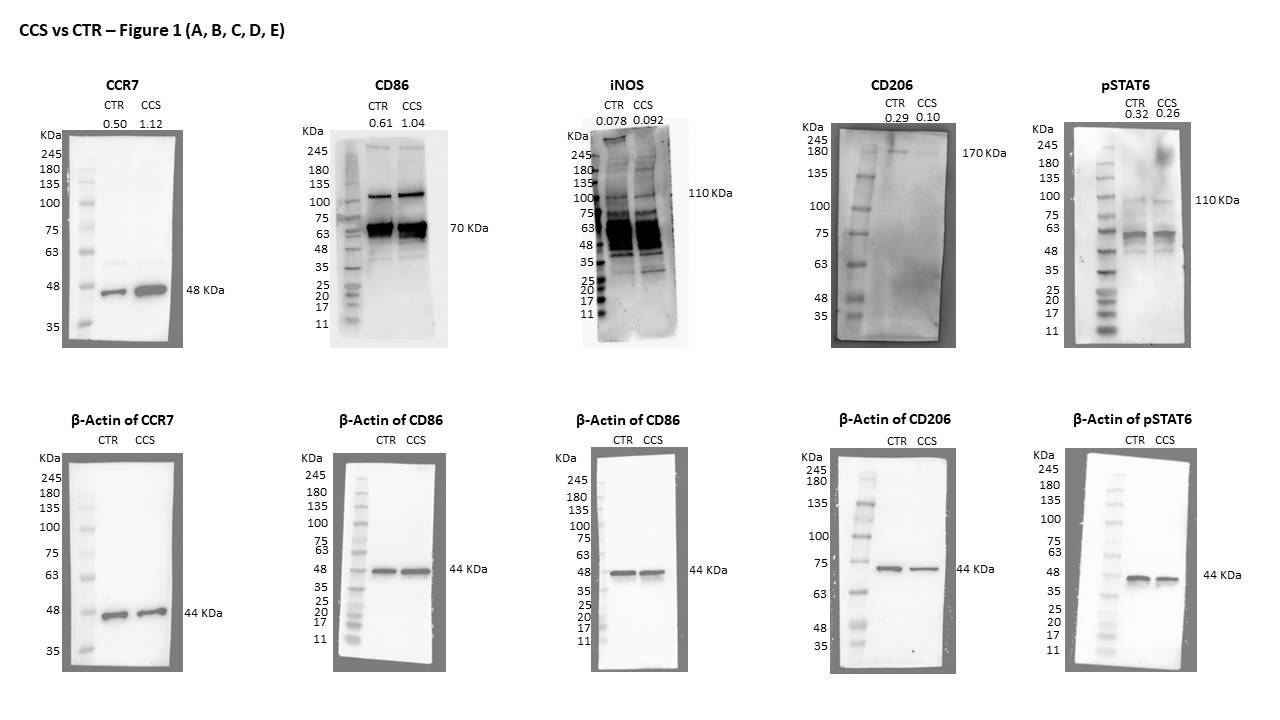

Supplement: Supplementary file 1 [file cancers-16-00714-s001.zip › Supplementary File S1/Uncropped Lenght Marker and Ratio_Figures/Uncropped Lenght Marker and Ratio_Figure 1.tif]

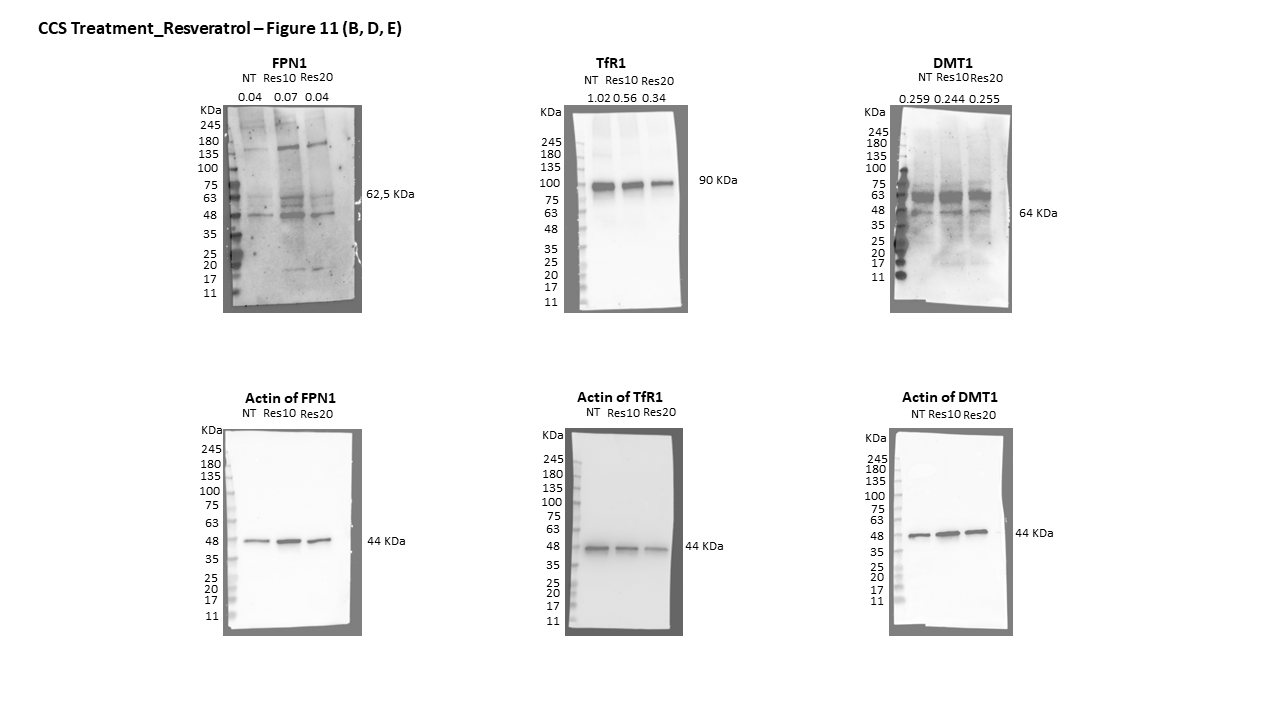

Supplement: Supplementary file 1 [file cancers-16-00714-s001.zip › Supplementary File S1/Uncropped Lenght Marker and Ratio_Figures/Uncropped Lenght Marker and Ratio_Figure 11.tif]

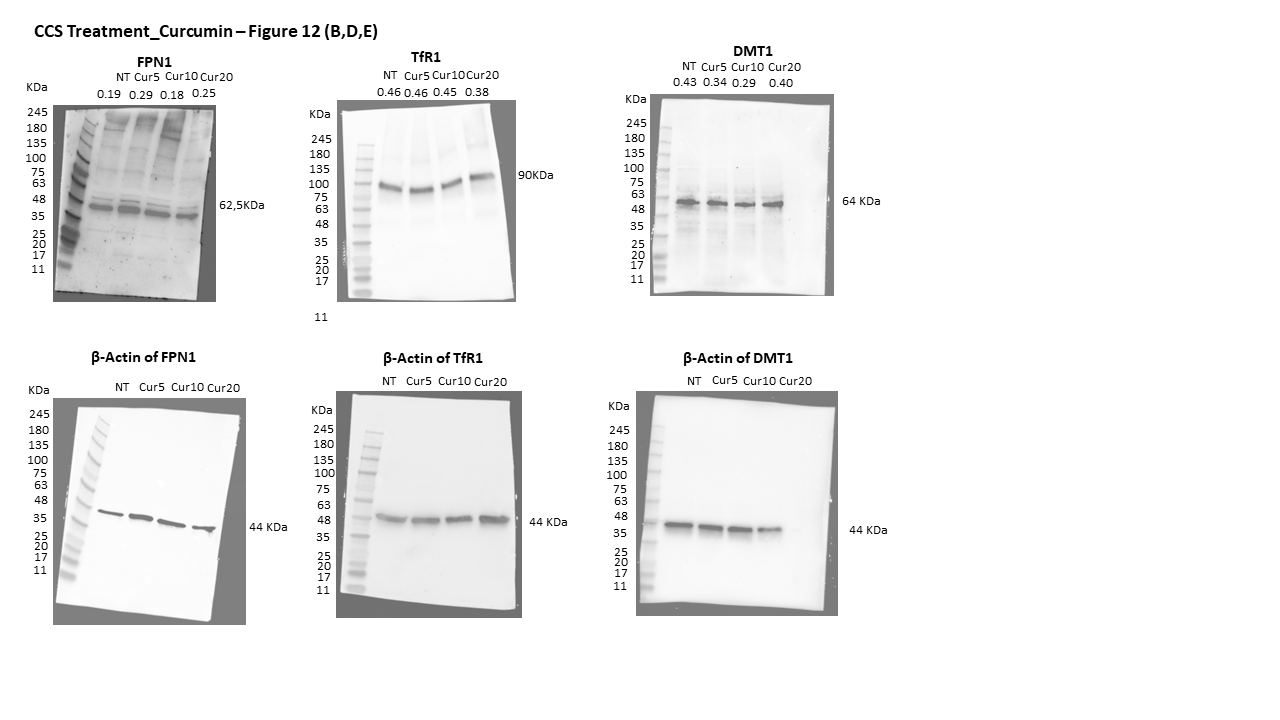

Supplement: Supplementary file 1 [file cancers-16-00714-s001.zip › Supplementary File S1/Uncropped Lenght Marker and Ratio_Figures/Uncropped Lenght Marker and Ratio_Figure 12.tif]

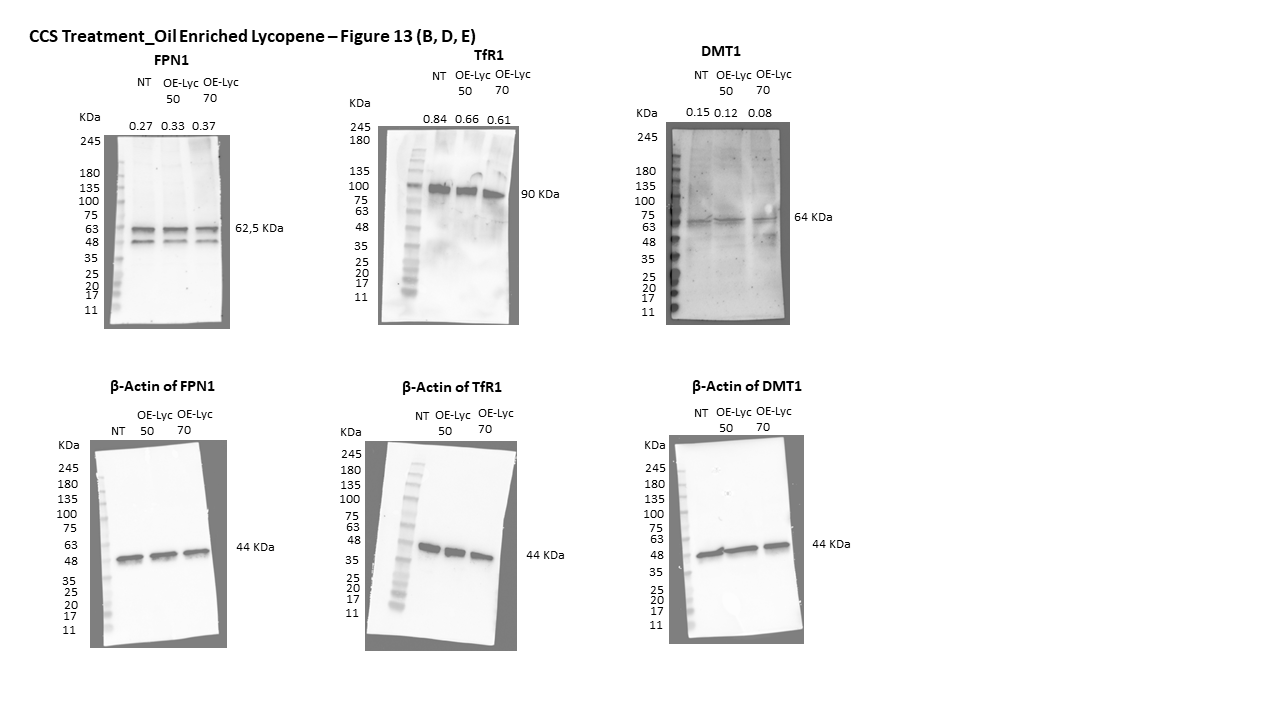

Supplement: Supplementary file 1 [file cancers-16-00714-s001.zip › Supplementary File S1/Uncropped Lenght Marker and Ratio_Figures/Uncropped Lenght Marker and Ratio_Figure 13.tif]

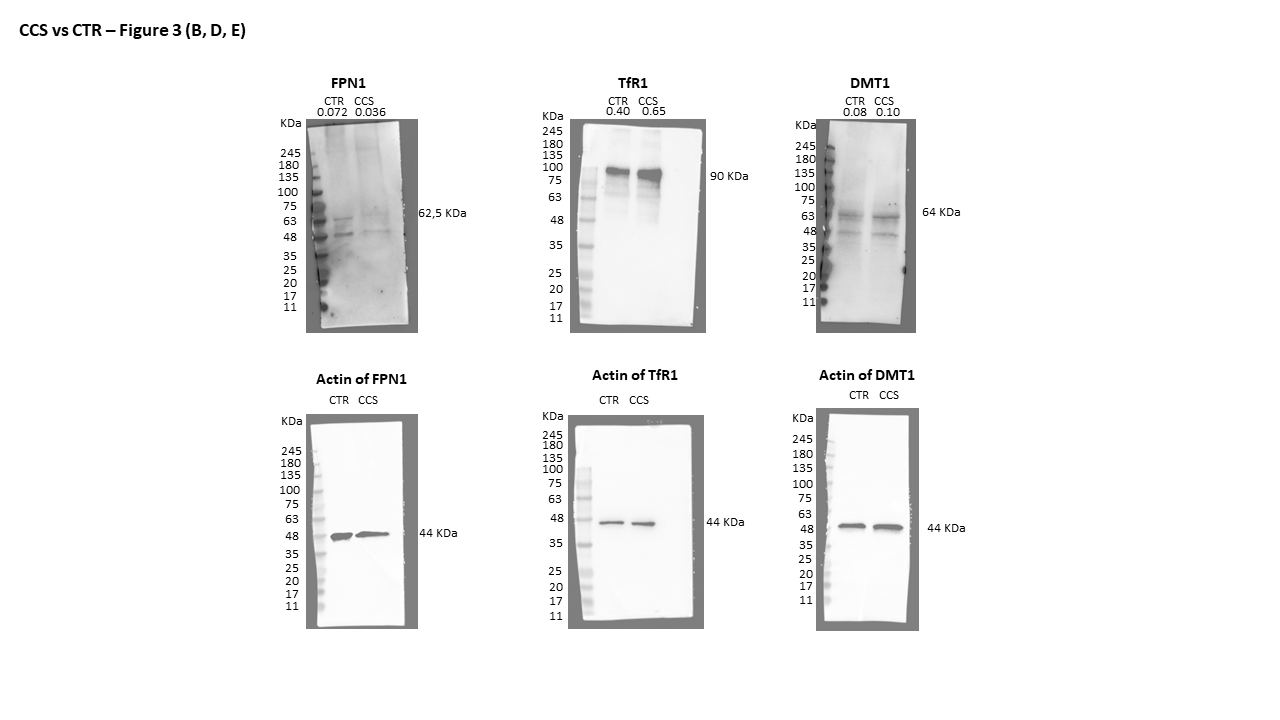

Supplement: Supplementary file 1 [file cancers-16-00714-s001.zip › Supplementary File S1/Uncropped Lenght Marker and Ratio_Figures/Uncropped Lenght Marker and Ratio_Figure 3.tif]

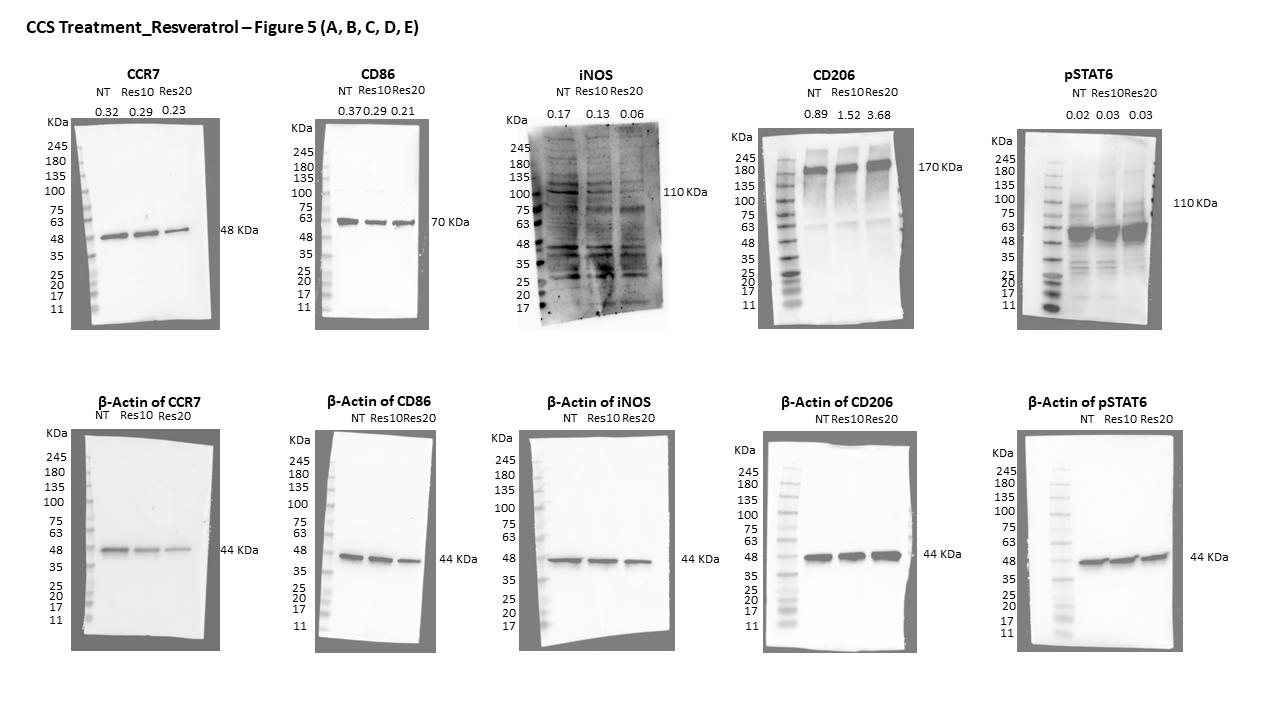

Supplement: Supplementary file 1 [file cancers-16-00714-s001.zip › Supplementary File S1/Uncropped Lenght Marker and Ratio_Figures/Uncropped Lenght Marker and Ratio_Figure 5.tif]

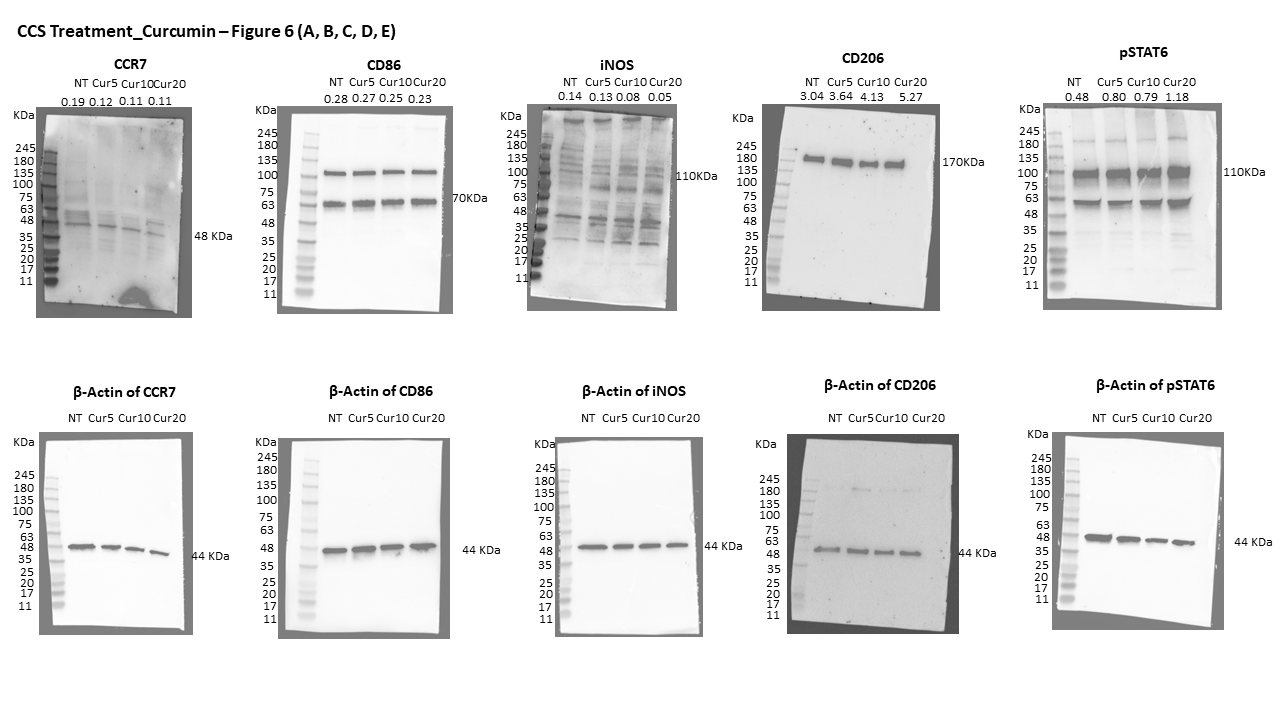

Supplement: Supplementary file 1 [file cancers-16-00714-s001.zip › Supplementary File S1/Uncropped Lenght Marker and Ratio_Figures/Uncropped Lenght Marker and Ratio_Figure 6.tif]

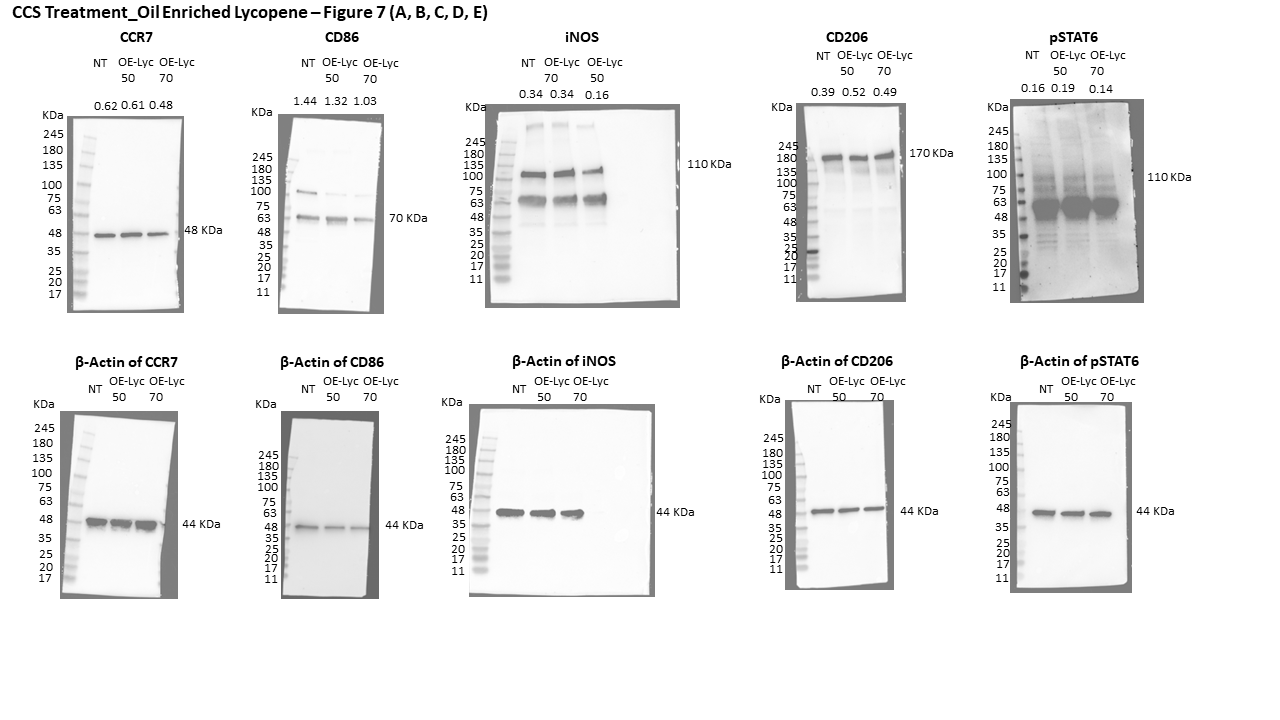

Supplement: Supplementary file 1 [file cancers-16-00714-s001.zip › Supplementary File S1/Uncropped Lenght Marker and Ratio_Figures/Uncropped Lenght Marker and Ratio_Figure 7.tif]

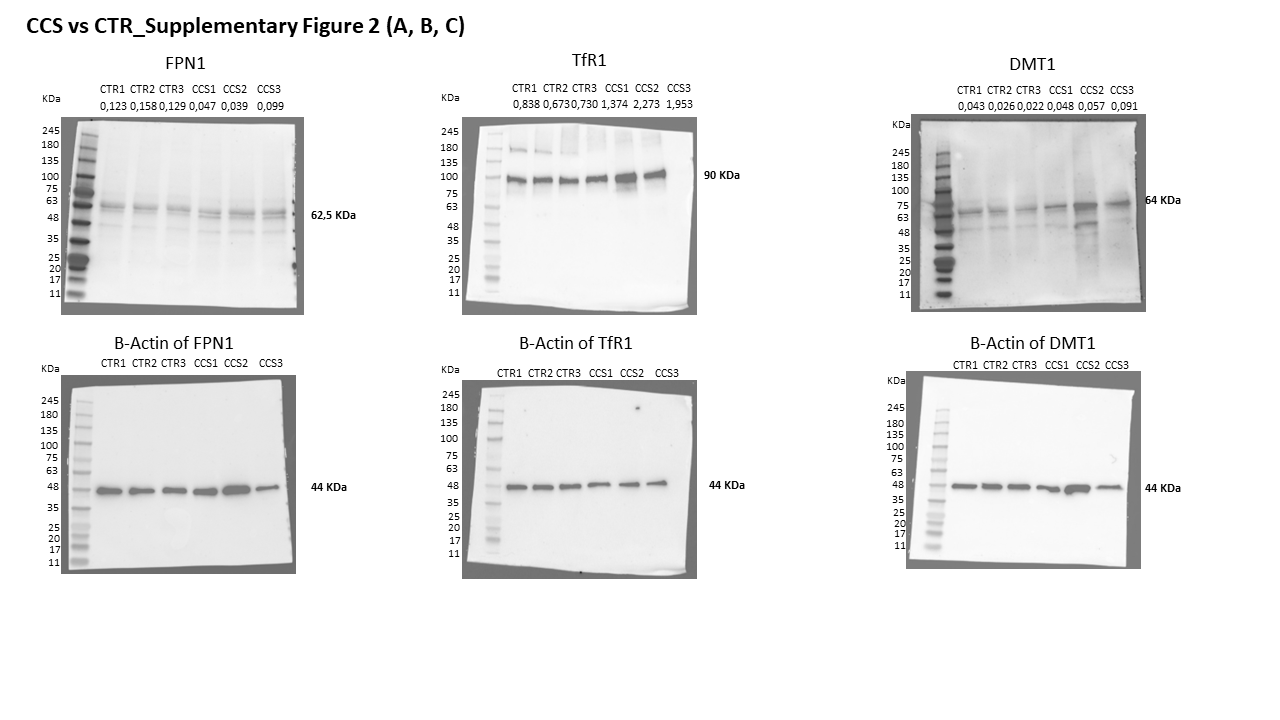

Supplement: Supplementary file 1 [file cancers-16-00714-s001.zip › Supplementary File S1/Uncropped Lenght Marker and Ratio_Supplementary Figures/Uncropped Lenght Marker and Ratio_Supplementary Figure 2.tif]

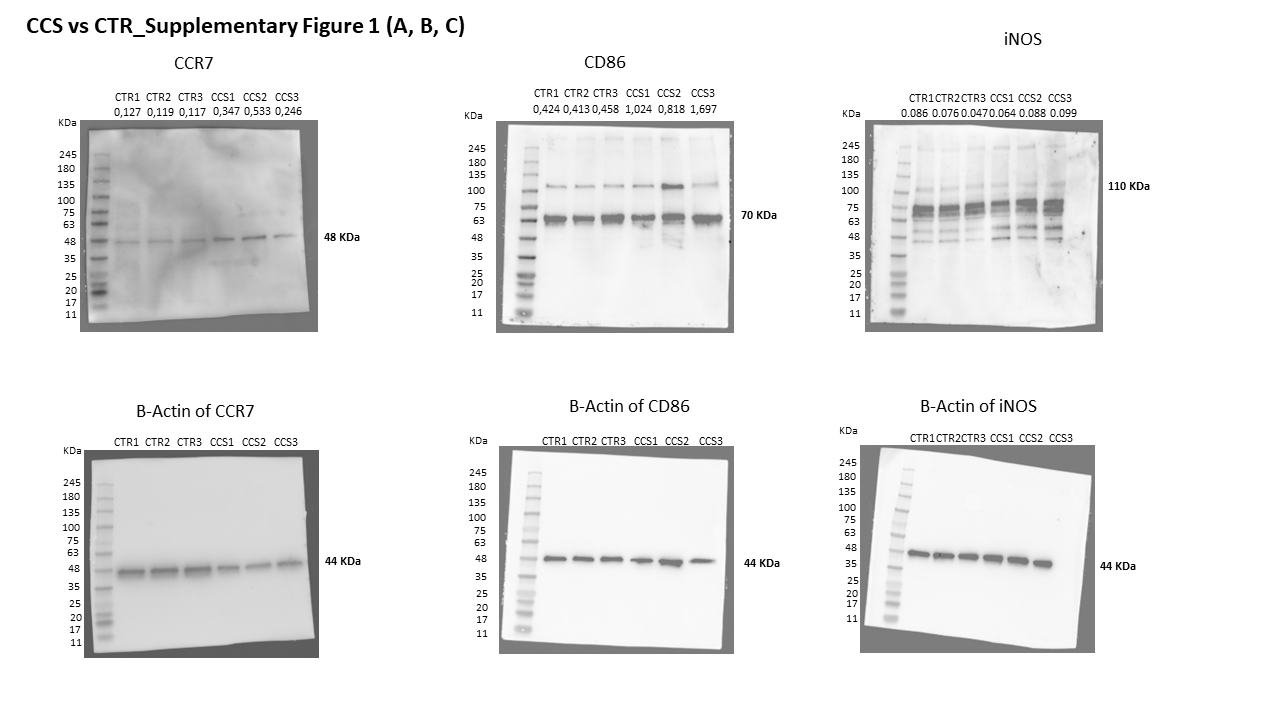

Supplement: Supplementary file 1 [file cancers-16-00714-s001.zip › Supplementary File S1/Uncropped Lenght Marker and Ratio_Supplementary Figures/Uncropped Lenght Marker and Ratio_Supplementary Figure1A,B,C.tif]

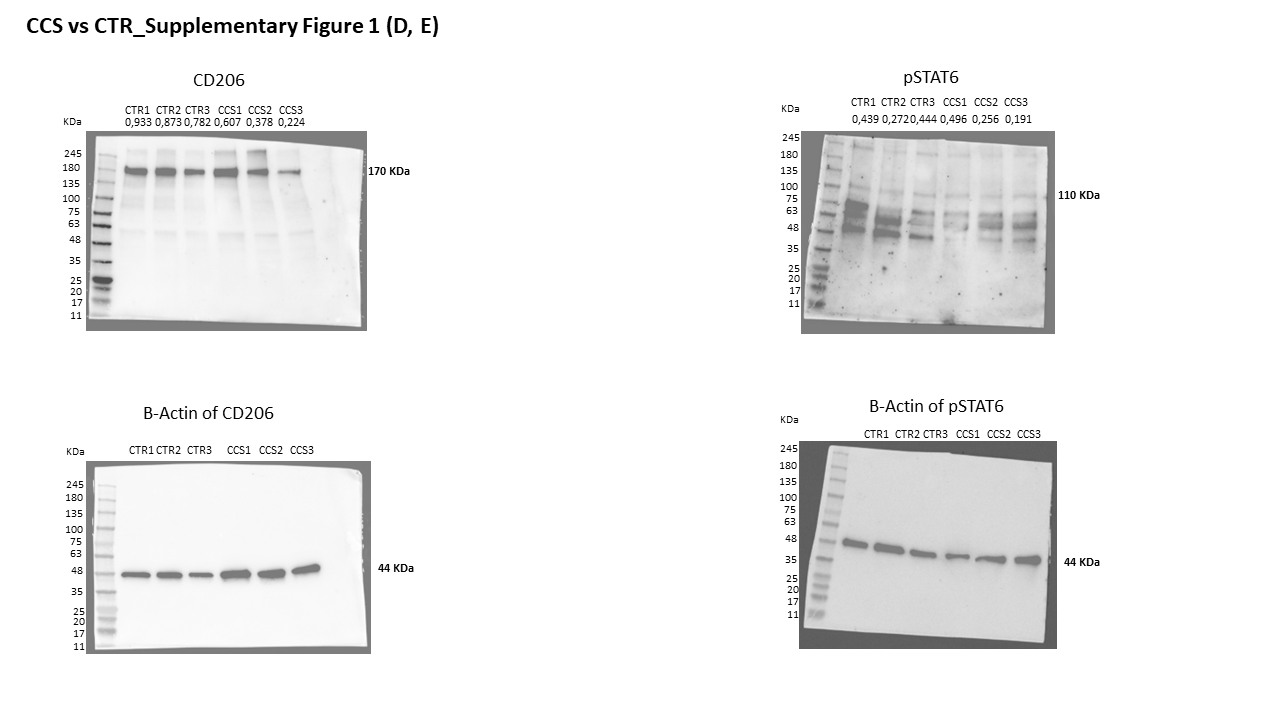

Supplement: Supplementary file 1 [file cancers-16-00714-s001.zip › Supplementary File S1/Uncropped Lenght Marker and Ratio_Supplementary Figures/Uncropped Lenght Marker and Ratio_Supplementary Figure1D,E.tif]
